# Lead in Air in Bangladesh: Exposure in a Rural Community with Elevated Blood Lead Concentrations among Young Children

**DOI:** 10.3390/ijerph15091947

**Published:** 2018-09-06

**Authors:** May K. Woo, Elisabeth S. Young, Md Golam Mostofa, Sakila Afroz, Md Omar Sharif Ibne Hasan, Quazi Quamruzzaman, David C. Bellinger, David C. Christiani, Maitreyi Mazumdar

**Affiliations:** 1 Department of Environmental Health, Harvard T.H. Chan School of Public Health, Boston, MA 02115, USA; david.bellinger@childrens.harvard.edu (D.C.B.); dchris@hsph.harvard.edu (D.C.C.); Maitreyi.MazumdarMDMPH@childrens.harvard.edu (M.M.); 2 Department of Social and Behavioral Sciences, Harvard T.H. Chan School of Public Health, Boston, MA 02115, USA; eyoung@hsph.harvard.edu; 3 Dhaka Community Hospital, Dhaka 1217, Bangladesh; mostofa07@gmail.com (M.G.M.); joya9878@yahoo.com (S.A.); dr.sharif0909@gmail.com (M.O.S.I.H.); dcht87@gmail.com (Q.Q.); 4 Department of Neurology, Boston Children’s Hospital, Boston, MA 02115, USA

**Keywords:** lead, exposure, Bangladesh, air pollution

## Abstract

Previous evaluations of a birth cohort in the Munshiganj District of Bangladesh had found that over 85% of 397 children aged 2–3 years had blood lead concentrations above the United States Centers for Disease Control and Prevention’s reference level of 5 μg/dL. Studies in urban areas of Bangladesh have found elevated levels of lead in the air due to industries and remaining contamination from the historic use of leaded gasoline. Sources of lead in rural areas of Bangladesh remain unknown. We conducted air sampling in both residential and industrial sites in Munshiganj to determine whether children are exposed to elevated lead concentrations in the air and study the association between the children’s blood lead levels and sampled air lead concentrations. Residential and industrial air samples in Munshiganj were found to have elevated lead concentrations (mean 1.22 μg/m^3^) but were not found to be associated with the observed blood lead concentrations. Lead in air is an important environmental health exposure risk to the for children in Munshiganj, and further research may shed light on specific sources to inform exposure prevention and mitigation programs.

## 1. Introduction

Lead is well established as an environmental health problem due to its neurotoxic effects in both children and adults [1,2,3]. Children are especially vulnerable to the neurotoxic effects of lead exposure due to their developmental state, body weight, and behaviors that increase risk of exposures [4]. Exposures in childhood have been shown to have significant long-term ramifications on both neurological and cognitive health and socioeconomic status over the life course [5,6,7]. The negative impacts of exposures on cognitive decline and social mobility make it essential to mitigate lead exposures, particularly for at-risk populations. Lead poisoning rates have declined in the United States and other developed countries as a result of awareness campaigns and the phasing out of lead from gasoline, paints, and consumer products. However, childhood lead exposure remains an issue in developing countries, such as Bangladesh, where epidemiological investigations have found high blood lead concentrations in children in and near the industrial center of Dhaka [8,9,10]. Children in rural communities of Bangladesh have also been found to have elevated blood lead concentrations [11]. Exposure in these communities may be due to continued use of leaded gasoline and older automobiles; local industries of ceramics, battery recycling, and mining; and other lifestyle characteristics, such as food items and product materials [12,13,14,15,16,17].

Testing in 397 children aged 2–3 years from a birth cohort in the Munshiganj District of Bangladesh found that over 85% had blood lead concentrations above the Centers for Disease Control and Prevention’s (CDC) current reference level of 5 μg/dL [18,19]; nearly 35% of these had concentrations above the previous CDC action level of 10 μg/dL [11,19]. Previous exposure assessments in and near these children’s homes found lead contamination of turmeric spice and food storage cans, but lead was undetected or detected at typical background levels in water, indoor dust, soil, cookware, and rice [20,21]. However, turmeric alone is unlikely to explain the elevated blood lead levels observed in the children. Inhalation of air is another possible route of exposure because suspended airborne lead particulates emitted from industrial and residential sources or resuspended from dust can enter the respiratory system, deposit in the lung, and absorb into the bloodstream [22]; however, this exposure route is yet to be measured in Munshiganj. Previous air pollution studies in Dhaka have found lead levels in ambient air ranging from 0.12 to 0.58 μg/m^3^ in particulate matter [23,24,25] and 74 μg/m^3^ in road dust [26]. These ambient levels are near to or higher than the United States National Ambient Air Quality Standard (NAAQS) for lead of 0.15 μg/m^3^ [27]. Due to the location of the Munshiganj District—only 50 km south of Dhaka city center—regional air pollution traveling from Dhaka and local emissions sources of lead may contribute to the elevated blood lead concentrations observed in the children.

To develop a comprehensive childhood lead poisoning prevention program in a community already known to be affected by lead poisoning, the identification of exposure sources is crucial. The objective of this study is twofold: to perform an air exposure assessment to measure and analyze ambient lead concentrations near children’s homes in Munshiganj, Bangladesh; and to identify spatial associations between these air lead exposures and observed blood lead concentrations, including the use of available geocoded data, on potential point sources of lead air emissions.

## 2. Materials and Methods

### 2.1. Study Population and Clinical Measurements

This research was conducted in Sirajdikhan Upazila, a primarily rural and agricultural region in the Munshiganj District of Bangladesh. Participants were children enrolled in an ongoing prospective birth cohort study investigating the associations between prenatal and postnatal arsenic, manganese, and lead exposures and reproductive birth outcomes and neurodevelopmental effects. Subject recruitment from areas in which Dhaka Community Hospital (DCH) operates rural health clinics was performed during 2008–2011. More information on the recruitment strategy, eligibility criteria, and questionnaire and sample collection from pregnant women and their newborn children has been described previously [28].

When children were aged 2–3 years, parents were re-contacted and invited for a follow-up visit in which venous whole blood samples were taken from children and analyzed for blood lead levels (BLLs) using a dynamic reaction cell inductively coupled plasma mass spectrometer (DRC-ICP-MS, DRC II, Perkin Elmer) [11,29].

Data from questionnaires completed at enrollment and follow-up visits were used to gather information on demographics and other covariates of the population. Trained interviewers conducted structured questionnaires to collect sociodemographic, medical, environmental, and geographic information from the participants. Covariates from the questionnaires that were selected for the present analysis were determined through review of the literature on their association with blood lead concentrations and/or risk of environmental exposures to lead [30,31,32,33,34]. The covariates included the sex and age of the child, maternal educational attainment, and geographic coordinates of the drinking well reported by the mother, with drinking well coordinates used as a proxy for the location of residence. Occupation was not adjusted for due to the large number of participants whose paternal occupations were reported as “other” (29%) and the uneven distribution of occupations across remaining categories.

Analysis for the current study was limited to those recruited by the Sirajdikhan clinic who participated in the 2–3 years follow-up. Of these 397 participants, 391 had reported drinking well coordinates. Of these, six were excluded due to missing demographic data (age or maternal education status), resulting in a final study population of 385 participants. A minimum sample size of 242 was estimated prior to the analysis for 80% statistical power and multiple regression study with four predictors. This study was approved by the Institutional Review Boards of the Harvard T.H. Chan School of Public Health and Boston Children’s Hospital and the Ethics Review Board of Dhaka Community Hospital.

### 2.2. Environmental Sample Collection and Analysis

#### 2.2.1. Sampling Site Selection

Geocoded coordinates of wells used for drinking water by each cohort member, as well as his or her BLL, was plotted in ArcGIS 10.3 [35]. Site selection for air sampling was performed using modified random selection of candidate areas across the approximately 15 km^2^ study area. The study area was divided into grids consisting of 1 km by 1 km grid cells. As sampling was to be performed at participants’ homes, grid cells that did not include any participant coordinates were excluded. Of those grid cells, four were chosen using number assignment and random number generation. A modified random site selection was chosen to minimize potential bias of unknown geographic attributes and the potentially uneven distribution of other spatial risk factors for elevated blood lead levels.

Within each of the four selected 1 km^2^ grid cells, two residential sites were selected, determined by participant availability and access. In addition, two industrial sites (a ceramics house and a battery manufacturing facility) were chosen based on research personnel’s knowledge of local industries for a total of ten sampling sites.

**Figure 1:**
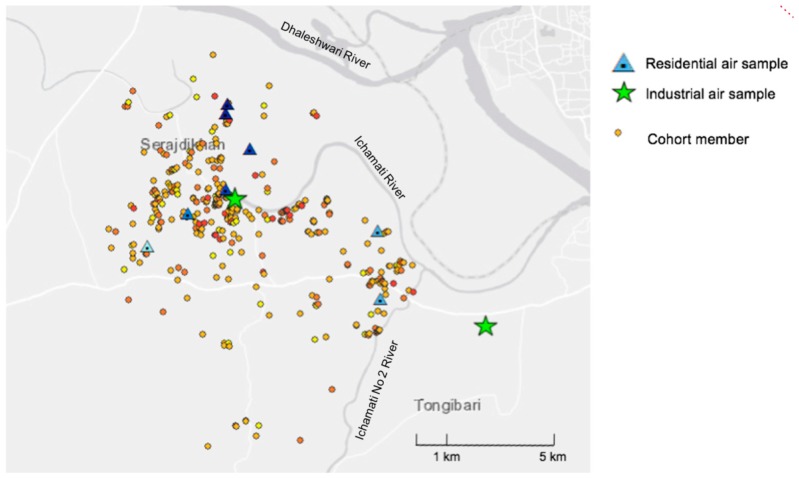
Geographical distribution of participants and air sampling sites.

Figure 1 shows the geographical distribution of air samples and cohort members.

#### 2.2.2. Air Sampling

Air sampling was performed over three days in January 2018 in Munshiganj. Total suspended particle (TSP) samples were taken using a Leland Legacy Air Sampler (SKC Inc., Eighty Four, PA, USA) with 37 mm, 2 μm pore size polytetrafluoroethylene (PTFE) filters (Pall Corporation, New York, NY, USA). Each sample was taken for 8 h during the daytime hours of approximately 10:00 AM to 6:00 PM, with constant airflow rate of 7 L/min.

At both residential and industrial sites, trained personnel set up the filters to be placed outdoors and connected via airflow tubes that passed through window openings to the air pumps, which were set up indoors for access to power and security. Filter faces were oriented such that airflow surrounding the filter was unobstructed; filters faced outward at approximately 4–7 feet in height from ground level. Site visits included observations of visibility of a road from sampling location and traffic level of the road, level of urbanization, and recording of global positioning system (GPS) coordinates of each sampling site, which was measured using Open Data Kit on Android devices [36].

Air flow and sampling duration were measured and confirmed before and after each sampling run. Two blank samples were used as field blanks. All samples were stored at room temperature and sent back to Boston, MA, USA, for analysis.

#### 2.2.3. Laboratory Analysis

Total suspended particle mass was determined by weighing the filters before and after exposure using an electronic microbalance (Mettler MT-5, Columbus, OH, USA) after allowing the samples to equilibrate to the controlled temperature and relative humidity of the balance room. A laboratory blank was used for validation of pre- and post-weighed measurements. Mass measurements were performed in duplicate or triplicate to ensure error within 0.005 mg.

Energy dispersive X-ray fluorescence (EDXRF) spectrometry was used to calculate the elemental composition of the air samples. EDXRF is a technique widely applied to the elemental analysis of ambient particles collected on filter media, with EPA method IO-3.3 specifying the protocol and the calibration procedure for analyzing elements on Teflon filters. EDXRF has been demonstrated to have improved accuracy and precision in comparison with different XRF analysis techniques [37]. Elemental analysis was conducted using an Epsilon 5 EDXRF spectrometer (PANalytical, Almelo, The Netherlands) at Harvard T.H. Chan School of Public Health (HSPH). This spectrometer utilizes secondary excitation from ten secondary selectable targets and employs a 600-W dual (scandium/tungsten, Sc/W) anode X-ray tube, a 100-kV generator, and a solid-state germanium (Ge) detector. The Epsilon 5′s limit of detection for lead is 0.009 μg.

### 2.3. Secondary Data Collection

To measure each participant’s proximity to traffic and industrial sources, geocoded data was obtained from publicly available databases. OpenStreetMap^®^ [38] contains geocoded map data on a variety of point, line, and area characteristics by country or region and includes roadway and other traffic data and land use characterizations. Google Maps API is a developer interface that can be used to search within the Google Maps database by distance radius, place type, or keyword [39] and can be searched for industries without the typical constraints of language or keyword searches. From these two databases, available geocoded data were compiled for the following points of interest type: traffic-related sources (identified using place type and keyword searches for roadways, gas stations, parking lots or structures, and bus and transit stations) and industrial sources (identified using place type and keyword searches for mills, ceramics houses, battery recycling or manufacturing shops, print shops, textile factories, packaging centers, and marketplaces). The numbers of traffic-related sources and industrial sources within a 1000 m radius buffer of the participants’ coordinates were used as a measure of traffic and industrial proximity. Buffer distances of 250 m, 500 m, and 2500 m were also analyzed according to the example set by previous traffic-related air pollution studies [40,41]. Due to the rural nature of the area, the 1000 m radius was used as the main buffer of analysis.

Road data was obtained from the Local Government Engineering Department (LGED) of Bangladesh [42]. Road types available in the study region included national highway, regional highway, zila (district) road, upazila road, union road, and village roads. For this analysis, highways, zila roads, and upazila roads were considered major roadways. The lengths of major roadways crossing the area within the 250 m, 500 m, 1000 m, and 2500 m radius buffers of the participants’ coordinates were calculated and used as the measure for traffic proximity.

### 2.4. Statistical Analyses—Ordinary Least Squares Regression

The air lead concentrations were plotted in ArcGIS based on their GPS coordinates. Samples taken within each of the four residential site grid cells were compared to each other using a two-sample *t*-test assuming unequal variances. The air lead concentration at each participant’s residence was then estimated using inverse squared distance weighting of the eight sampled residential air lead concentrations.

Descriptive statistics for blood lead concentrations (BLLs) and the selected subject characteristics (age, sex, maternal education) were calculated. Normality of the BLL outcome was assessed using histograms and normal Q-Q plots, which suggest that BLL distribution was close to normal and the Central Limit Theorem should be applied with our sample size of 385. Outlier sensitivity was analyzed by removing two participants with BLLs over 1.5 times the interquartile range of BLLs for the population (BLL of 36.26 and 39.88 μg/dL).

A multivariate regression model was built to evaluate the association between the participant’s interpolated air lead concentrations and his or her BLL. Demographic covariates included in the model (age, sex, and maternal education) were based on scientific evidence as described above. BLL was treated as a continuous outcome variable. Age at the 20–40 months clinical visit was modeled as a continuous variable (months) and sex as a binary variable. Maternal education was modeled as a binary variable (secondary education or more, primary education or less). The covariate of interest was the participant’s estimated air lead exposure interpolated using inverse squared distance weighting of the sample concentrations.

BLL = β1 + β2×(Age) + β3×(Sex) + β4×(Maternal Education) + β5×[Pb_air_] (1)

Multivariate regression models were fit in order to analyze the association between source proximity and BLL. BLL, the continuous outcome, was modeled for the association with length of major roadways within a 1000 m buffer (continuous) and number of industrial point sources within a 1000 m buffer (continuous). The adjusted model included sex, age, and maternal education and included both traffic and industrial sources simultaneously due to their likely relationship, i.e., areas of higher roadway density would be expected to have a greater number of industrial establishments.

### 2.5. Statistical Analysis—Spatially Filtered Regression

Ordinary least squares (OLS) regression methods, while straightforward and easily interpreted, have two major assumptions: normality and homoscedasticity of the residuals. While the diagnostics met these assumptions, the inherently geographic nature of the data does not fit the third assumption of OLS run on geographic data, which states that model residuals must not be spatially autocorrelated. Space not taken into account in the model may lead to model misspecification, resulting in invalid effect estimates and errors [43,44,45].

The residuals of the OLS linear regression model indicated statistically significant spatial autocorrelation (Moran’s I Z-score 0.102, *p* < 0.01). Thus, spatial patterns in the blood lead levels were explored using optimized hot spot analysis, which identifies statistically significant spatial clusters of high values (hot spots) and low values (cold spots) of blood lead levels based on the Getis-Ord Gi(d) statistic.

Spatial filtering, a method that removes the spatial component of variables within a regression, was applied to study the relationship between air lead, demographic covariates, and blood lead after controlling for the spatial effect. The spatial filtering method utilizes the Getis-Ord Gi(d) statistic to determine critical distances at which there is no further spatial autocorrelation for each covariate and uses these distances to filter out the spatial effect [46]. Since the original OLS model used the interpolated air lead covariate based on inverse squared distance weighting, this covariate was inherently spatially autocorrelated. Thus, for this spatial analysis, the nearest air lead measurement was assigned as air lead exposure for each cohort member (splitting up the study area into Thiessen polygons). The filtering distances for air lead concentrations, age, sex, and maternal education were 150 m, 650 m, 650 m, and 150 m, respectively. The final regression model incorporated the filtered and spatial components of each covariate as described by the following equation:

BLL = β1 + β2(Age_filtered_) + β3(Age_spatial_) + β4(Sex_filtered_) + β5(Sex_spatial_) + β6(Maternal Education_filtered_) + β7(Maternal Education_spatial_) + β8[Pb_air, filtered_] + β9[Pb_air, spatial_] (2)

Model diagnostics were run on the resulting filtered model using Moran’s I univariate test of the model residuals to determine whether there was remaining inherent spatial autocorrelation after filtering for the measured covariates.

All statistical analyses were performed in R version 3.3.1 [47].

## 3. Results

We used the demographic and BLL data available from 385 of the original 397 study participants. Chi-square and Wilcoxon sign rank tests comparing the study population with those excluded due to missing data indicated no statistically significant differences. The demographic characteristics of the study population are presented in Table 1. BLLs at age 2–3 years ranged from 0.005–39.88 μg/dL, with a mean of 9.074 μg/dL. In our study population, 86% of the children had BLLs above the CDC reference level of 5 μg/dL, and 35% were above 10 μg/dL.

The concentrations of lead and total suspended particles measured in the air samples are presented in Table 2. The lead concentrations in the eight residential air samples had a mean of 1.22 μg/m^3^, with a range of 0.14–3.10 μg/m^3^. The air sample taken from the battery manufacturing plant was found to be elevated, with a lead concentration of 376.58 μg/m^3^. All samples had concentrations of lead that were elevated compared to the field blank samples simultaneously analyzed in EDXRF. The comparison between the two samples taken within each of the sampling site grid cells indicated no statistically significant difference, suggesting similarity of residential air lead concentrations between samples that were near each other (two sample *t*-test assuming unequal variances, *t* = 0.327, *p* = 0.75).

The air lead concentration exposures estimated for each participant’s coordinates by inverse squared distance weighting of the eight residential samples produced a mean air lead concentration of 0.96 μg/m^3^ across the 385 participants, with a range of 0.16–2.93 μg/m^3^. Estimated air concentrations for participant coordinates are summarized in Table 3.

The multivariate regression for blood lead levels and inverse squared distance weighted air lead concentrations—adjusted for age, sex, and maternal education—found no statistically significant association for any covariate. The effect estimate of air lead concentration of 1.0 μg/m^3^ on BLL (μg/dL) was −0.40 (95% confidence interval (CI) −1.04, 0.34). Similar results were obtained for the study population excluding the two outliers with BLLs above 36 μg/dL, which were outside of 1.5 times the interquartile range of BLLs for the population. These results are summarized in Table 4A.

**Figure 2:**
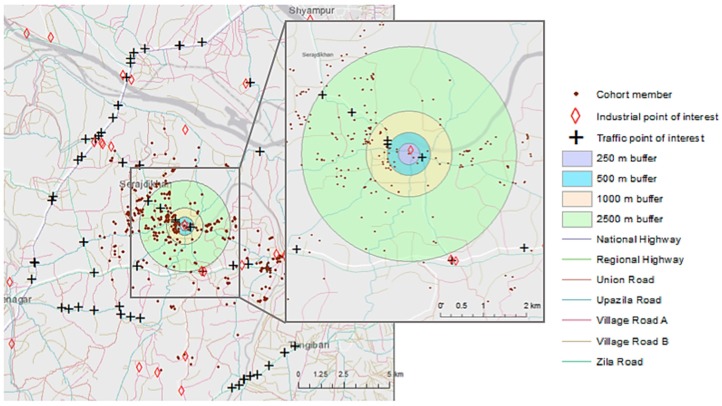
Geographical distribution of identified industries, traffic point and line sources, and a visualization of the 250 m, 500 m, 1000 m, and 2500 m buffers surrounding each cohort member in which sources were indexed.

The number of point sources within 1000 m of the participant’s coordinates ranged from zero to four (mean of 0.89) for traffic-related points and zero to three (mean of 0.33) for industry-related points. The length of major roadways within the 1000 m buffer ranged from 0–6.35 km (mean of 2.05 km). No highways were within 1000 m of any of the participant’s coordinates. Because of the overlap between traffic point source identification and roadways as traffic proximity indicators, only the roadway length measure was included in the regression model for source proximity. Figure 2 provides a visualization of the spatial distribution of traffic and industrial points of interest, roadways, and buffers.

The multivariate regression for blood lead levels and traffic and industrial proximity—adjusted for age, sex, and maternal education—found no statistically significant associations for any of the covariates. The effect estimate of number of industrial points of interest on BLL (μg/dL) was −0.01 (95% CI −0.64, 0.62), and the effect estimate of length of major roadways was −0.00008 (95% CI −0.00047, 0.0003). Similar results were obtained for the study population excluding the two outliers as well as for the industries and road length measures within the 250 m, 500 m, and 2500 m buffers. The results are summarized in Table 4B.

**Figure 3:**
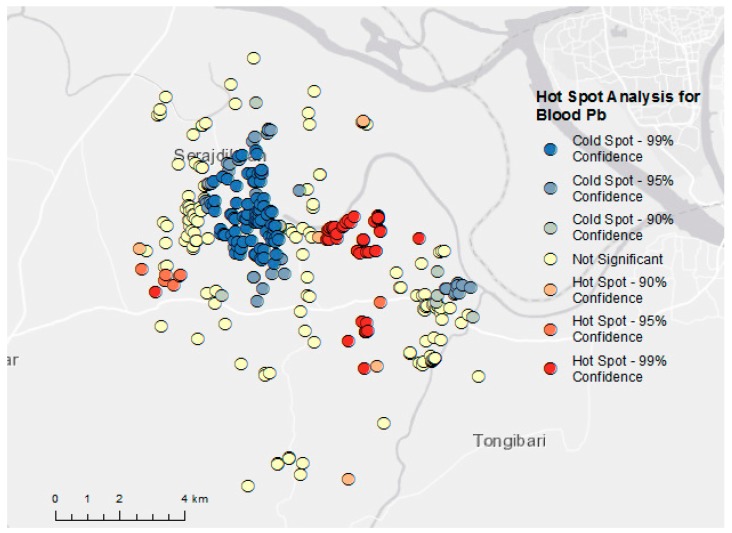
Hot spot analysis using Getis Ord-Gi(d) statistic indicating hot and cold spots and their confidence levels for cohort member blood lead levels.

Exploration of the OLS model’s residuals indicated statistically significant spatial autocorrelation (Moran’s I = 0.102, *p* < 0.001), demonstrating a violation of an assumption of OLS and the subsequent need for a spatial regression. Results from the hot spot analysis indicated statistically significant hot and cold spots for blood lead levels (see Figure 3), demonstrating the need to account for space in the regression analysis.

The spatially filtered model results, summarized in Table 5, indicate that after filtering for space, neither the air lead concentration covariate nor the demographic covariates were associated with the observed blood lead levels. Moran’s I univariate test, indicated by the z(I) value for each filtered and spatial covariate, shows the effectiveness of the filtering method in teasing out the spatial autocorrelation for each covariate.

A test for spatial autocorrelation of the resulting model Moran’s I for the filtered model was 0.098 (*p* < 0.05), indicating that there is still some spatial autocorrelation not accounted for by the measured covariates in our model. All results presented are spatial models run for the cohort excluding the two high BLL outliers (N = 383).

## 4. Discussion

In the present study, we found all but one sample of total suspended particles taken at residential sites in Munshiganj, Bangladesh to be above the EPA NAAQS 3-h ambient air quality standard of 0.15 μg/m^3^ for lead, with a mean concentration of 1.22 μg/m^3^ and range of 0.14–3.10 μg/m^3^. The mean concentration was above the WHO guideline of 0.5 μg/m^3^ (annual average) for lead in air [48]. These concentrations are above the lead concentrations of approximately 0.12–0.58 μg/m^3^ observed in previous air pollution studies in Dhaka [23,24,49] from the late 1990s to early 2000s when leaded gasoline was still in the process of being phased out. The detection of air lead concentrations in our samples above these levels indicates that lead in air is an issue of similar magnitude despite the fact that rural, agricultural areas such as Munshiganj would expect to have fewer emissions sources.

The estimated air lead concentrations at each child’s coordinates were not associated with their BLLs in analyses, including the full study population as well as the population excluding the two high BLL outliers. This may be due to the small sample size of air samples from which estimates were derived. While there are other ambient air estimation and modeling techniques that are widely used and validated, such as land use regression and air dispersion modeling of local and regional sources, the inputs necessary for these models were unavailable for the study area. We also did not find a significant association between the children’s proximity to major roadways and industries and their BLLs in analyses utilizing buffer distances of 250 m, 500 m, 1000 m, and 2500 m. Further model diagnostics and spatial analyses demonstrated the need to include space into the model because participant and environmental data were geographic in nature. Despite our spatial regression model’s failure to determine a significant predictor for these observed BLLs, the significant hot and cold spots of BLLs suggests the likely presence of an unmeasured spatial factor in our analysis; this is supported by similar findings in a previous study by Forsyth et al. [21] in rural Bangladesh.

The residential air lead concentrations found in the present study pose an important health risk to residents, particularly children, in Munshiganj. The neurodevelopmental effects of lead exposure on children have been extensively studied, with research demonstrating that there is no safe level of lead [50]. Without exposure mitigation, resident exposures to these elevated concentrations in the long term may continue to lead to neuropsychiatric disorders, IQ deficiencies, and reproductive and developmental health effects.

However, effective exposure mitigation requires the identification of exposure sources, which proves to be especially challenging in areas with a large informal sector. As reported by the Bangladesh Bureau of Statistics, Sirajdikhan—the administrative subregion where a majority of the cohort members was located—has the largest percentage of cottage industries compared to other upazilas in the Munshiganj District [51]. The high lead concentrations in the industrial air samples of the present study suggest the contribution of these cottage industry emissions to the air lead levels in Munshiganj. One such sample taken at a battery manufacturing plant measured a concentration of 376.6 μg/m^3^, which was similar in magnitude to the environmental measurements of 340–430 μg/m^3^ reported in previous assessments of lead acid battery plants in developing countries [52,53]. As the motor vehicle industry grows in Bangladesh, the demand for lead acid batteries will increase, adding to the importance of small-scale recycling and manufacturing plants [14]. The lack of engineering and other controls for lead emissions from the informal sector, including but not limited to the battery industry, poses health risks to both workers and the surrounding general population, thereby highlighting the continued need for environmental and occupational health and safety programs and research in developing countries [15,16,54,55,56].

There are many limitations to consider in the current study. The small sample size of eight residential air samples and two industrial samples lacks high geographic resolution and distribution across the study area. Considering the high variability of air pollution within an airshed, creating an estimation surface from eight samples can be expected to have considerable uncertainty. The samples were also taken at one snapshot in time, and therefore they lack information on the impact of temperature, weather, and seasonality on the measured air lead concentrations. Furthermore, while these samples were taken for total suspended particles, it is not representative of the inhalable size fraction. The analysis involving road and industrial proximity is also subject to much uncertainty due to the lack of available data on industries in Munshiganj. The combination of the lack of available information on the locations, sectors, and size of these cottage industries and their importance on lead emissions, as evidenced in the present study, may cause biases in the geocoded points included in the analysis. The model of the association between estimated air lead concentrations and BLLs was performed using multivariate linear regression with demographic covariates chosen a priori based on scientific literature. While it is possible that other statistical models may better fit the relationship, diagnostics of the data and its residuals did not indicate any trend. In addition, there may be unmeasured confounders or lead sources that were not included in our model. Furthermore, the use of drinking water well coordinates as residence may influence the clustering observed for the cohort members. Finally, a major limitation in our study was the discord between the temporality of exposure and outcome. The sampled air concentrations were used as a proxy for past air concentrations, which assumed that current environmental conditions and their distribution are similar to past conditions. Considering the short (one month) half-life of lead in blood [57], relating past exposures to current blood levels may not be appropriate.

Despite these limitations, this was the first residential air sampling for lead in rural Munshiganj, and one of few exposure assessments in this cohort’s study area that demonstrated detectable and elevated levels of lead. The levels of lead measured, which was comparable to the levels measured in Dhaka in the late 1990s, demonstrates the importance of air pollution as a source of exposure in the region, despite the latter being a largely rural and agricultural region. It demonstrates the need for further air pollution studies in the area and the need for better control of small-scale industries as emission sources as well for the protection of workers and the public from such sources. This supports previous research, which had indicated geographic heterogeneity of blood lead levels and exposures in the region [21].

Avenues for future research in this study area include further air monitoring for lead utilizing a greater sample size with wider geographical distribution that encompasses both residential and industrial sites. In addition, using the spatial analyses performed in this study and predictive probabilities of lead exposure by location will prove helpful for future study design elements such as sampling site selection, which may oppose or refine the effect estimates obtained in the current study. A more systematic and detailed indexing of geocoded industries and other point sources in the region would also help model source attribution for lead exposures in these children. Other sources of lead to be studied—both in the air and through other routes of exposure—may include roofing and wall sheet material [13], e-waste recycling methods [58], and parental occupation in the lead industry [59]. Furthermore, including other individual-level covariates that may influence blood lead levels, such as nutritional status, would also be of interest.

## 5. Conclusions

This study adds to the literature demonstrating the need for research and more detailed documentation on lead and other environmental health hazards in developing countries and rural populations that considers the variations in exposure sources and pathways in these settings. A database of informal sectors of lead mining, smelting, battery manufacturing, and other cottage industries will be important in evaluating high exposure sources in both urban and rural areas. Further air sampling will be needed to inform targeted emissions and future exposure prevention strategies in this study population.

## Figures and Tables

**Table 1:** Selected characteristics of the study population of children in Munshiganj, Bangladesh (*N* = 385).

Characteristics		No. (%)	Mean ± SD
Age at follow-up	22–26 months 27–31 months 32–36 months	167 (43.4) 168 (43.6) 50 (12.9)	28.23 ± 2.94
Sex	Male Female	202 (52.5) 183 (47.5)	
Maternal education	Primary or lower Secondary or more	190 (49.4) 195 (50.6)	
Blood lead level (μg/dL)	0–5 μg/dL 5–10 μg/dL 10–15 μg/dL 15–20 μg/dL >20 μg/dL	52 (13.5) 199 (51.7) 101 (26.2) 24 (6.23) 9 (2.34)	9.074 ± 4.50

**Table 2:** Concentrations for lead (Pb) and total suspended particles (TSP) measured by energy dispersive X-ray fluorescence (EDXRF) spectrometry in 8-h samples (7 L/min flow rate) collected in Munshiganj, Bangladesh, in January 2018. Samples taken within the same sampling site grid cell are indicated by site identifier A–D.

Sample and Site Type	Pb μg/m^3^ (SD)	TSP μg/m^3^
Residential A	2.04 (0.021)	370.24
Residential A	1.37 (0.017)	589.29
Residential B	0.14 (0.010)	243.15
Residential B	0.21 (0.011)	209.23
Residential C	3.10 (0.027)	440.48
Residential C	2.50 (0.023)	325.89
Residential D	0.19 (0.011)	308.63
Residential D	0.20 (0.011)	322.91
Industrial, battery manufacturing	376.58 (2.141)	910.12
Industrial, ceramics house	0.47 (0.012)	511.31
Blank	0.03 (0.009)	2.083
Blank	0.02 (0.009)	0.102

**Table 3:** Air lead concentrations for each participant’s coordinates estimated by inverse squared distance weighting. The United States Environmental Protection Agency’s National Ambient Air Quality Standard for lead in total suspended particles is 0.15 μg/m^3^.

Estimated Air Lead Concentration (μg/m^3^)	No. of Study Participants
<0.15 (NAAQS)	0
0.16–0.5	104
0.6–1.0	120
1.1–1.5	101
>1.5	60

**Table 4:** Multivariate regression results for (A) the model evaluating the relationship between blood lead levels (BLLs) and air lead concentrations, interpolated by inverse squared distance weighting and adjusted for age, sex, and maternal education; and (B) the model evaluating the relationship between BLLs and number of industrial points of interest and length of major roadways within 250 m, 500 m, 1000 m, and 2500 m buffers of the residences.

**A. Blood Lead Levels and Estimated Air Lead**								
	**Full Dataset (*N* = 385)**				**Excluding 2 Outliers (*N* = 383)**			
	**Coefficient**	**SE**	***t*-Value**	***p*-Value**	**Coefficient**	**SE**	***t*-Value**	***p*-Value**
Intercept	10.39	2.31	4.49	<0.001	9.94	2.04	4.86	<0.001
Estimated air Pb (μg/m^3^)	−0.40	0.38	−1.04	0.30	−0.31	0.34	−0.91	0.36
Age (months)	−0.04	0.08	−0.45	0.66	−0.04	0.07	−0.53	0.59
Sex (female)	0.16	0.46	0.35	0.73	−0.439	0.41	1.08	0.28
Maternal education (primary or less)	0.31	0.46	−0.68	0.50	0.61	0.41	−1.49	0.14
	Adjusted R^2^ = −0.0047				Adjusted R^2^ = 0.0028			
**B. Blood Lead Levels and Traffic and Proximity**								
	**Full Dataset (*N* = 385)**				**Excluding 2 Outliers (*N* = 383)**			
	**Coefficient**	**SE**	***t*-Value**	***p*-Value**	**Coefficient**	**SE**	***t*-Value**	***p*-Value**
Intercept	10.44	2.32	4.49	<0.001	9.99	2.06	4.86	<0.001
No. of industrial sources within 1000 m	−0.01	0.32	−0.02	0.98	−0.14	0.31	−0.46	0.64
Length of major roads within 1000 m	−0.00008	0.0002	−0.53	0.59	0.00002	0.0001	0.19	0.85
Age (months)	−0.04	0.08	−0.59	0.56	−0.05	0.07	−0.74	0.46
Sex (female)	0.21	0.47	0.44	0.66	0.48	0.41	1.16	0.25
Maternal education (primary or less)	0.32	0.47	−0.75	0.45	−0.61	0.41	−1.48	0.14
	Adjusted R^2^ = −0.01				Adjusted R^2^ = −0.001			

**Table 5:** Spatially filtered regression results for the model evaluating the relationship between blood lead levels (BLLs) and air lead (Pb) concentrations, estimated for each cohort member by its nearest residential air sample and adjusted for age, sex, and maternal education.

Covariate	Coefficient	SE	*t*-Value	*p*-Value	Moran’s I Z-Score
Intercept	10.19	6.26	1.63	0.10	
Air Pb_filtered_	−3.41	4.14	−0.83	0.41	−0.104
Age_filtered_	0.06	0.18	0.34	0.73	0.11
Sex_filtered_	0.24	0.43	0.54	0.58	−0.117
Maternal education_filtered_	1.83	2.04	0.90	0.36	−0.05
Air Pb_spatial_	−0.27	0.23	−1.16	0.25	1.03 *
Age_spatial_	−0.07	0.07	−0.93	0.35	0.82 *
Sex_spatial_	1.93	1.08	1.78	0.08	0.416 *
Maternal education_spatial_	0.34	0.60	0.57	0.57	−0.05

Adjusted R-squared: 0.00306. Univariate Moran’s I of model residuals, prefiltering = 0.102, *p* < 0.01; Univariate Moran’s I of model residuals, postfiltering = 0.09, *p* < 0.05. * = statistically significant spatial autocorrelation (Univariate Moran’s I test).
